# Supplementary material for: Past COVID-19: The Impact on IVF Outcomes Based on Follicular Fluid Lipid Profile
Source: Int J Mol Sci. 2022 Dec 20;24(1):10. doi: 10.3390/ijms24010010 (PMC9820085; doi:10.3390/ijms24010010)
Supplement: Supplementary file 1 [file ijms-24-00010-s001.zip › ijms-2004931-supplementary.pdf]

Supplementary materials.

Past COVID-19: the impact on IVF outcomes based on follicular fluid lipid profile

Lomova N.A.[1], Dolgushina N.V.[1,2], Tokareva A.O.[1], Chagovets V.V[1], Starodubtseva N.L.[1,3], Kulikov I.A.[1,2], Sukhikh G.T.[1], Frankevich V.E.[1,4]

[1] National Medical Research Center of Obstetrics, Gynecology and Perinatology the name of Academician V.I. Kulakov, 117198 Moscow, Russia.

[2] Department of Obstetrics, Gynecology, Perinatology and Reproductology, Institute of professional education, Federal State Autonomous Educational Institution of Higher Education I.M. Sechenov First Moscow State Medical University of the Ministry of Health of the Russian Federation (Sechenov University), 119435 Moscow, Russia.

[3] *Moscow Institute of Physics and Technology, 141700 Moscow, Russia.*

[4] *Siberian State Medical University, 634050 Tomsk, Russia*

*Correspondence: vfrankevich@gmail.com

Table S1. Lipid levels characterizing differences in history of SARS-COV-2

Lipid	No COVID-19	Mild COVID-19	Severe COVID-19	p-value
Positive ion mode				
CE 18:3	8.26e5(6.84e5;1.03e6)	8.16e5(6.62e5;9.7e5)	9.1e5(7.71e5;1.16e6)	0.03
PC 16:0_16:0	6.92e5(5.88e5;8.04e5)	6.51e5(5.08e5;7.52e5)	7.36e5(6.4e5;8.37e5)	0.003
PC 16:0_18:1	7.74e6(5.96e6;9.65e6)	7.29e6(5.79e6;9e6)	8.31e6(7.24e6;9.76e6)	0.03
PC 16:0_18:2	2.98e7(2.4e7;3.5e7)	2.67e7(2.28e7;3.23e7)	3.05e7(2.5e7;3.6e7)	0.05
PC 16:0_20:4	1.3e7(1.08e7;1.55e7)	1.17e7(9.84e6;1.45e7)	1.39e7(1.18e7;1.63e7)	0.01
PC 18:0_20:3	1.87e6(1.22e6;2.93e6)	1.67e6(9.99e5;2.68e6)	2.91e6(2.04e6;3.6e6)	<0.001
PC 18:0_20:4	7.34e6(5.07e6;9.68e6)	6.24e6(4.86e6;8.52e6)	9.06e6(6.61e6;1.09e7)	0.002
PC 18:0_20:5	1.94e6(1.61e6;2.39e6)	1.73e6(1.53e6;2.11e6)	2.07e6(1.72e6;2.39e6)	0.02
PC 18:1_18:2	8.5e6(7.09e6;1.05e7)	7.58e6(6.38e6;9.37e6)	9.55e6(7.71e6;1.04e7)	0.01
SM d20:5/24:0	4.76e5(3.12e5;8.17e5)	3.85e5(2.32e5;6.42e5)	5.51e5(3.73e5;8.02e5)	0.01
Negative ion mode				
LPC 16:0	4.53e6(3.94e6;5.06e6)	4.28e6(3.9e6;4.83e6)	4.65e6(4.31e6;5.17e6)	0.01
LPC 16:1	6.9e4(6.05e4;7.96e4)	6.97e4(5.39e4;8.23e4)	7.37e4(6.89e4;8.43e4)	0.02
LPC 18:0	1.75e6(1.53e6;2e6)	1.71e6(1.52e6;1.92e6)	1.85e6(1.7e6;2.17e6)	0.02
MGDG 18:0_20:3	2.56e5(1.32e5;3.73e5)	2.33e5(1.29e5;3.24e5)	3.15e5(2.13e5;4.21e5)	0.007
MGDG 18:1_18:1	1.12e6(9.11e5;1.35e6)	1.02e6(8.09e5;1.17e6)	1.22e6(1.04e6;1.41e6)	0.003
OxPE 18:0_18:2(OOO)	4.09e4(3.09e4;5.19e4)	3.49e4(2.71e4;4.98e4)	4.37e4(3.35e4;6.26e4)	0.02
OxPG 18:1_20:3(1O)	1.22e5(1.08e5;1.41e5)	1.18e5(1.02e5;1.35e5)	1.34e5(1.19e5;1.56e5)	0.010

PC 16:0_18:2	4.78e6(3.88e6;5.64e6)	4.19e6(3.52e6;5.12e6)	4.76e6(4.05e6;5.63e6)	0.012
PC 16:0_22:5	6.56e5(5.23e5;8.93e5)	5.77e5(4.98e5;7.12e5)	7.23e5(5.9e5;9.24e5)	0.011
PC 16:1_18:2	8.17e4(5.22e4;1.05e5)	7.33e4(5.62e4;9.65e4)	9.42e4(7.31e4;1.07e5)	0.04
PC 18:0_18:1	3.56e4(2.12e4;4.93e4)	2.84e4(1.58e4;4.25e4)	3.83e4(2.35e4;5.17e4)	0.03
PC 18:1_18:2	1.43e6(1.23e6;1.7e6)	1.29e6(1.1e6;1.58e6)	1.63e6(1.39e6;1.82e6)	<0.001
PC O-16:0/20:4	2.35e5(1.97e5;2.79e5)	2.21e5(1.91e5;2.6e5)	2.61e5(2.15e5;3.04e5)	0.005
PC O-16:1/20:4	1.85e5(1.43e5;2.24e5)	1.72e5(1.41e5;2.1e5)	2.03e5(1.62e5;2.45e5)	0.03
PE 18:0_18:2	9.14e4(6.73e4;1.1e5)	7.55e4(6.12e4;9.89e4)	8.78e4(6.99e4;1.18e5)	0.02
PE 18:0_20:4	7.19e4(4.93e4;9.39e4)	5.44e4(4.19e4;7.56e4)	7.65e4(6.39e4;1.03e5)	<0.001
PE O-16:0/20:4	4.95e4(3.85e4;6.35e4)	4.35e4(3.01e4;5.8e4)	6.26e4(4.35e4;7.97e4)	0.001
PE P-16:0/22:5	7.34e5(6.35e5;8.26e5)	6.65e5(5.84e5;7.67e5)	7.26e5(6.46e5;8.37e5)	0.02
PI 18:0_18:2	5.18e4(4.24e4;6.76e4)	4.87e4(3.76e4;5.68e4)	5.44e4(4.2e4;7.13e4)	0.03
SM d16:1/18:0	1.66e6(1.45e6;1.85e6)	1.52e6(1.3e6;1.72e6)	1.66e6(1.47e6;1.86e6)	0.02
SM d22:0/20:2	8.76e5(6.61e5;1.08e6)	8.13e5(6.11e5;9.84e5)	1e6(7.53e5;1.16e6)	0.04
SM d22:0/20:3	5.7e5(4.51e5;7.69e5)	5.48e5(4.29e5;6.64e5)	6.98e5(5.48e5;8.43e5)	0.005
SM d22:1/18:0	7.62e5(4.72e5;1.18e6)	6.98e5(3.88e5;1.03e6)	9.4e5(7.06e5;1.15e6)	0.03

Table S2. Lipid levels characterizing differences in FF in patients with no COVID-19 and mild COVID-19.

Lipid	No COVID-19	Mild COVID-19	p-value
Positive ion mode			
PC 16:0_16:0	6.92e5(5.88e5;8.04e5)	6.51e5(5.08e5;7.52e5)	0.01
PC 16:0_18:2	2.98e7(2.4e7;3.5e7)	2.67e7(2.28e7;3.23e7)	0.04
PC 18:1_18:2	8.5e6(7.09e6;1.05e7)	7.58e6(6.38e6;9.37e6)	0.02
SM d20:5/24:0	4.76e5(3.12e5;8.17e5)	3.85e5(2.32e5;6.42e5)	0.02
Negative ion mode			
MGDG 18:1_18:1	1.12e6(9.11e5;1.35e6)	1.02e6(8.09e5;1.17e6)	0.02
PC 16:0_18:2	4.78e6(3.88e6;5.64e6)	4.19e6(3.52e6;5.12e6)	0.01
PC 18:1_18:2	1.43e6(1.23e6;1.7e6)	1.29e6(1.1e6;1.58e6)	0.01
PE 18:0_18:2	9.14e4(6.73e4;1.1e5)	7.55e4(6.12e4;9.89e4)	0.01
PE 18:0_20:4	7.19e4(4.93e4;9.39e4)	5.44e4(4.19e4;7.56e4)	0.003
PE O-16:0/20:4	4.95e4(3.85e4;6.35e4)	4.35e4(3.01e4;5.8e4)	0.03
PE P-16:0/22:5	7.34e5(6.35e5;8.26e5)	6.65e5(5.84e5;7.67e5)	0.01
PI 18:0_18:2	5.18e4(4.24e4;6.76e4)	4.87e4(3.76e4;5.68e4)	0.03
SM d16:1/18:0	1.66e6(1.45e6;1.85e6)	1.52e6(1.3e6;1.72e6)	0.02

Table S3. Lipid levels characterizing differences in follicular fluid in asymptomatic patients and patients with severe COVID-19.

Lipid	No COVID-19	Severe COVID-19	p-value
Positive ion mode			
CE 18:3	8.26e5(6.84e5;1.03e6)	9.1e5(7.71e5;1.16e6)	0.03
LPC 16:1	2.85e5(2.5e5;3.27e5)	3.18e5(2.81e5;3.51e5)	0.02
PC 18:0_20:3	1.87e6(1.22e6;2.93e6)	2.91e6(2.04e6;3.6e6)	<0.001

PC 18:0_20:4	7.34e6(5.07e6;9.68e6)	9.06e6(6.61e6;1.09e7)	0.008
Negative ion mode			
LPC 16:1	6.9e4(6.05e4;7.96e4)	7.37e4(6.89e4;8.43e4)	0.006
LPC 18:0	1.75e6(1.53e6;2e6)	1.85e6(1.7e6;2.17e6)	0.04
MGDG 18:0_20:3	2.56e5(1.32e5;3.73e5)	3.15e5(2.13e5;4.21e5)	0.007
OxPG 18:1_20:3(1O)	1.22e5(1.08e5;1.41e5)	1.34e5(1.19e5;1.56e5)	0.04
PC O-16:0/18:1	6.11e4(2.52e4;8.27e4)	6.96e4(4.06e4;9.25e4)	0.05
PC O-16:0/20:4	2.35e5(1.97e5;2.79e5)	2.61e5(2.15e5;3.04e5)	0.04
PE O-16:0/20:4	4.95e4(3.85e4;6.35e4)	6.26e4(4.35e4;7.97e4)	0.02
SM d22:0/20:3	5.7e5(4.51e5;7.69e5)	6.98e5(5.48e5;8.43e5)	0.01

Table S4. Lipid levels characterizing differences in follicular fluid in patients with different forms of COVID-19.

Lipid	Mild COVID-19	Severe COVID-19	p-value
Positive ion mode			
CE 18:0	4.75e5(3.87e5;5.82e5)	5.49e5(4.42e5;6.35e5)	0.04
CE 18:3	8.16e5(6.62e5;9.7e5)	9.1e5(7.71e5;1.16e6)	0.009
CE 20:4	5.46e6(4.44e6;6.37e6)	6.00e6(4.85e6;7.09e6)	0.03
LPC 16:0	2.88e6(2.63e6;3.17e6)	3.01e6(2.81e6;3.46e6)	0.03
LPC 18:0	7.01e6(6.21e6;8.2e6)	7.7e6(6.65e6;8.95e6)	0.02
OxLPC 22:5(OH)	2.31e5(2.1e5;2.53e5)	2.42e5(2.26e5;2.75e5)	0.03
PC O-16:0/20:4	1.39e6(1.1e6;1.65e6)	1.65e6(1.19e6;1.99e6)	0.02
PC 16:0_16:0	6.51e5(5.08e5;7.52e5)	7.36e5(6.4e5;8.37e5)	0.002
PC 16:0_18:1	7.29e6(5.79e6;9e6)	8.31e6(7.24e6;9.76e6)	0.005
PC 16:0_18:2	2.67e7(2.28e7;3.23e7)	3.05e7(2.5e7;3.6e7)	0.03
PC 16:0_20:4	1.17e7(9.84e6;1.45e7)	1.39e7(1.18e7;1.63e7)	0.003
PC 16:0_20:5	2.95e6(2.55e6;3.64e6)	3.34e6(2.77e6;4.02e6)	0.02
PC 16:0_22:5	3.44e6(2.79e6;4.53e6)	4.11e6(3.28e6;5.19e6)	0.03
PC 16:1_18:2	4.27e5(3.42e5;6.03e5)	5.37e5(4.24e5;6.68e5)	0.01
PC 16:1_22:6	1.33e6(9.75e5;1.61e6)	1.56e6(1.15e6;1.82e6)	0.01
PC 18:0_18:1	1.75e6(1.36e6;2.22e6)	2.09e6(1.61e6;2.47e6)	0.02
PC 18:0_18:2	1.5e7(8.41e6;1.81e7)	1.76e7(1.13e7;2.12e7)	0.02
PC 18:0_20:3	1.67e6(9.99e5;2.68e6)	2.91e6(2.04e6;3.6e6)	<0.001
PC 18:0_20:4	6.24e6(4.86e6;8.52e6)	9.06e6(6.61e6;1.09e7)	<0.001
PC 18:0_20:5	1.73e6(1.53e6;2.11e6)	2.07e6(1.72e6;2.39e6)	0.006
PC 18:1_18:2	7.58e6(6.38e6;9.37e6)	9.55e6(7.71e6;1.04e7)	0.003
SM d16:0/16:1	3.93e5(3.46e5;4.84e5)	4.47e5(3.98e5;5.3e5)	0.04
SM d18:1/22:0	1.74e6(3.86e5;2.89e6)	2.49e6(8.76e5;4.52e6)	0.04
SM d18:2/16:0	8e5(7.24e5;9.76e5)	8.84e5(7.87e5;1.04e6)	0.03
SM d18:2/24:1	1.5e6(1.09e6;2.1e6)	1.79e6(1.2e6;2.98e6)	0.05
SM d20:5/24:0	3.85e5(2.32e5;6.42e5)	5.51e5(3.73e5;8.02e5)	0.006
Negative ion mode			
LPC 16:0	4.28e6(3.9e6;4.83e6)	4.65e6(4.31e6;5.17e6)	0.002
LPC 16:1	6.97e4(5.39e4;8.23e4)	7.37e4(6.89e4;8.43e4)	0.03
LPC 18:0	1.71e6(1.52e6;1.92e6)	1.85e6(1.7e6;2.17e6)	0.005

LPC 18:1	6.81e5(6.17e5;8.37e5)	7.79e5(6.71e5;9.07e5)	0.025
LPC 20:4	2.93e5(2.42e5;3.81e5)	3.21e5(2.83e5;4.35e5)	0.04
LPC P-16:0	4.13e4(3.6e4;4.96e4)	4.77e4(3.75e4;5.38e4)	0.03
MGDG 18:0_20:3	2.33e5(1.29e5;3.24e5)	3.15e5(2.13e5;4.21e5)	0.003
MGDG 18:1_18:1	1.02e6(8.09e5;1.17e6)	1.22e6(1.04e6;1.41e6)	0.001
OxPC 18:2_16:1(OO)	2.82e5(2.22e5;3.7e5)	3.39e5(2.61e5;3.99e5)	0.03
OxPE 18:0_18:2(OOO)	3.49e4(2.71e4;4.98e4)	4.37e4(3.35e4;6.26e4)	0.007
OxPG 18:1_20:3(1O)	1.18e5(1.02e5;1.35e5)	1.34e5(1.19e5;1.56e5)	0.003
PC 16:0_18:2	4.19e6(3.52e6;5.12e6)	4.76e6(4.05e6;5.63e6)	0.01
PC 16:0_22:5	5.77e5(4.98e5;7.12e5)	7.23e5(5.9e5;9.24e5)	0.003
PC 16:1_18:2	7.33e4(5.62e4;9.65e4)	9.42e4(7.31e4;1.07e5)	0.009
PC 18:0_18:1	2.84e4(1.58e4;4.25e4)	3.83e4(2.35e4;5.17e4)	0.009
PC 18:0_18:2	2.24e6(1.6e6;2.95e6)	2.66e6(2e6;3.54e6)	0.02
PC 18:1_18:2	1.29e6(1.1e6;1.58e6)	1.63e6(1.39e6;1.82e6)	0.000
PC O-16:0/20:4	2.21e5(1.91e5;2.6e5)	2.61e5(2.15e5;3.04e5)	0.001
PC O-16:1/20:4	1.72e5(1.41e5;2.1e5)	2.03e5(1.62e5;2.45e5)	0.009
PC P-18:1/20:4	9.78e4(6.08e4;1.27e5)	1.18e5(8.28e4;1.44e5)	0.04
PE 18:0_18:2	7.55e4(6.12e4;9.89e4)	8.78e4(6.99e4;1.18e5)	0.02
PE 18:0_20:4	5.44e4(4.19e4;7.56e4)	7.65e4(6.39e4;1.03e5)	<0.001
PE O-16:0/20:4	4.35e4(3.01e4;5.8e4)	6.26e4(4.35e4;7.97e4)	<0.001
PE P-16:0/22:5	6.65e5(5.84e5;7.67e5)	7.26e5(6.46e5;8.37e5)	0.020
PI 18:0_18:2	4.87e4(3.76e4;5.68e4)	5.44e4(4.2e4;7.13e4)	0.02
SM d16:1/18:0	1.52e6(1.3e6;1.72e6)	1.66e6(1.47e6;1.86e6)	0.02
SM d18:4/20:4	2.32e4(1.83e4;2.9e4)	2.66e4(2.3e4;3.06e4)	0.010
SM d22:0/20:2	8.13e5(6.11e5;9.84e5)	1.00e6(7.53e5;1.16e6)	0.009
SM d22:0/20:3	5.48e5(4.29e5;6.64e5)	6.98e5(5.48e5;8.43e5)	0.001
SM d22:1/18:0	6.98e5(3.88e5;1.03e6)	9.4e5(7.06e5;1.15e6)	0.006
SM d22:6/18:2	7.13e4(5.72e4;8.37e4)	7.82e4(6.83e4;8.72e4)	0.03
SM d22:6/20:3	3.39e4(2.71e4;7.61e4)	6.07e4(3.12e4;8.66e4)	0.04
SM d24:1/18:0	2.77e5(2.06e5;3.31e5)	3.09e5(2.71e5;3.47e5)	0.04
